# Acute Lumbar Pyogenic Spondylitis With Multiple Abscesses Complicated by a Septic Shock That Required Emergency Surgical Open Drainage: A Case Report

**DOI:** 10.7759/cureus.34844

**Published:** 2023-02-10

**Authors:** Takumi Kurita, Masaki Tatsumura, Fumihiko Eto, Toru Funayama, Masashi Yamazaki

**Affiliations:** 1 Department of Orthopaedic Surgery and Sports Medicine, Tsukuba University Hospital Mito Clinical Education and Training Center, Mito Kyodo General Hospital, Mito, JPN; 2 Department of Orthopaedic Surgery, Faculty of Medicine, University of Tsukuba, Tsukuba, JPN

**Keywords:** a lethal case, open drainage, a case report, successful treatment, emergency surgery, septic shock, epidural abscess, intramuscular abscesses, pyogenic spondylitis, acute phase

## Abstract

We treated a patient with pyogenic spondylitis complicated by septic shock, who was saved by emergency surgery.

The patient was a 75-year-old man with back pain, fever, and weakness in the lower limbs four days before. Upon admission to our hospital, he had tachycardia, tachypnea, fever, and fluctuating vital signs. His quick sequential organ failure assessment (SOFA) score was 2. Emergent magnetic resonance imaging showed scattered intramuscular abscesses and an epidural abscess. Gram-positive cocci were detected in a blood sample. He was diagnosed with pyogenic spondylitis complicated by sepsis. Intravenous antimicrobial therapy with cefepime, vancomycin, and clindamycin was added. However, he developed tachycardia and hypotension three hours after arrival at our hospital, so he received a blood transfusion and noradrenaline and underwent emergent surgical open drainage since percutaneous drainage was difficult to perform because of scattered abscesses. Paralysis of the proximal lower extremities was recovered after surgery. Postoperatively, the causative organism was found to be methicillin-susceptible *Staphylococcus aureus* and intravenous antimicrobial therapy for 81 days. Three years after surgery, the patient remains free of recurrence with improvement in the activity of daily living to the extent that he could walk.

The outcome of our patient suggests that surgery may be a lifesaving measure in cases whose uncontrollable vital signs by pyogenic spondylitis are complicated by sepsis. Preoperative judgment is extremely important in difficult-to-control cases because surgical invasion can be lethal.

## Introduction

The number of patients with pyogenic spondylitis has increased in recent years. The reasons for the increase in such patients include an aging population, lifestyle-related diseases, and drug-induced immunodeficiencies [1]. The basic treatment strategy for pyogenic spondylitis consists of broad-spectrum antimicrobial agents given early during the diagnostic workup, with reviewing and de-escalation of the agents based on the results of blood cultures. In most cases, conservative treatment should be effective. If it is not effective, percutaneous drainage or surgery may be used. The surgical option is commonly used after the patient has stable vital signs because vital signs are unstable in the acute phase of septic shock [2]. In this report, we describe a patient in the acute stage of pyogenic spondylitis complicated by septic shock, who received life-saving emergency surgery. Some physicians may abandon emergency surgery for such systemic infectious diseases because it may not be life-saving. We hope that they will be encouraged by our successful experience, which we hope will lead to saving lives.

## Case presentation

**Figure 1:**
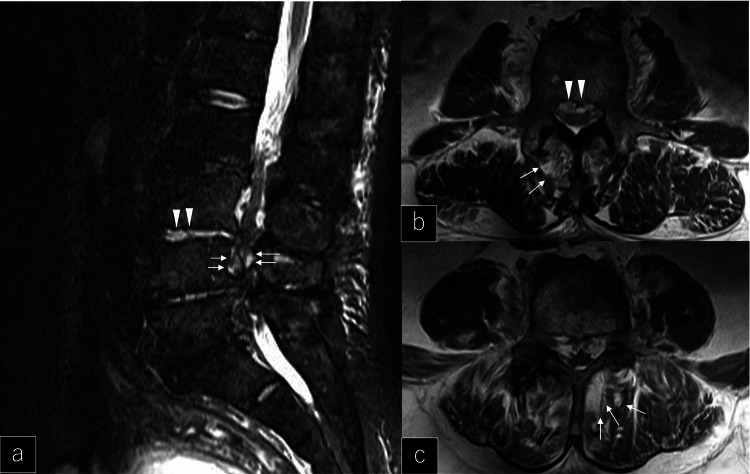
Preoperative lumbar magnetic resonance imaging (MRI). a: Sagittal slice Magnetic resonance imaging (MRI) showed intradiscal edema (white arrowheads) and epidural edema that were considered abscesses and compressed the dural sac (white arrows). b, c: T2-weighted image of axial slice. MRI showed scattered intramuscular edema (white arrows) and epidural edema that were considered abscesses and compressed the dural sac (white arrowheads).

The patient was a 75-year-old man with an unremarkable past medical history. He had no history of diabetes and was in good health with no regular medication prior to this pyogenic spondylitis complicated by sepsis. He had been admitted to a previous hospital with chief complaints of back pain, fever, and weakness of the lower limbs. Four days after admission he developed problems with defecation and was referred to our hospital because an intestinal obstruction was suspected. Upon admission to our hospital, the patient was disorientated, with tachycardia at 130 beats/min, tachypnea at 34 breaths/min, fever of 38.7°C, and fluctuating vital signs. His quick sequential organ failure assessment (SOFA) score was 2. Physical examination revealed loss of the rectoanal reflex, motor paralysis of the lower extremities, and grade 2 on the manual muscle test (MMT). The results of blood tests showed the following: white blood cells 23,300 cells/μL, C-reactive protein 35.7 mg/dL, and lactate 4.14 mMol/L. The patient was considered critical because of the severe inflammatory findings. His platelet count was low (67,000 cells/μL), and his coagulation capacity was slightly decreased (prothrombin time international normalized ratio 1.14 and activated partial thromboplastin time 43.0 sec) (Table 1). Emergent lumbar magnetic resonance imaging (MRI) showed intradiscal edema, scattered intramuscular edema near the spinous processes, and epidural edema which were considered abscesses (Figure 1a-c). Gram-positive cocci were detected in a blood sample.

**Table 1:** Results of blood testing at the time of admission. The results of blood tests showed the following: white blood cells 23,300 cells/μL, C-reactive protein 35.7 mg/dL, and lactate 4.14 mMol/L. The patient was considered critical because of the severe inflammatory findings. His platelet count was low (67,000 cells/μL), and his coagulation capacity was slightly decreased (prothrombin time international normalized ratio 1.14 and activated partial thromboplastin time 43.0 sec).

Category	Test	Unit	Result	Reference Values
Biochemical Blood Test	Albumin	(g/dL)	1.7	3.9-
	Urea Nitrogen	(mg/dL)	52	8.0-22
	Creatinine	(mg/dL)	1.3	-1.2
	Sodium	(mEq/L)	140	136-147
	Chloride	(mEq/L)	101	98-109
	Potassium	(mEq/L)	3.8	3.6-5.0
	Creatine Kinase	(IU/L)	2,455	57-197
	C-reactive Protein	(mg/dL)	35.7	-0.3
Blood Cell Count	White Blood Cell	(cells/μL)	23,300	3,100-8,400
	Hemoglobin	(g/dL)	13.4	13.1-16.3
	Platelet Count	(cells/μL)	67,000	145,000-329,000
Coagulation Test	Prothrombin Time International Normalized Ratio		1.14	0.90-1.15
	Activated Partial Thromboplastin Time	(sec)	43.0	20-40
Blood Gas	pH		7.514	7.35-7.45
	Partial Pressure of Carbon Dioxide	(mmHg)	30.7	35-45
	Partial Pressure of Oxygen	(mmHg)	63.3	75-
	Hydrogen carbonate Ion	(mMol/L)	24.2	22-26
	Lactate	(mMol/L)	4.14	0.4-1.6

Altogether, the patient’s findings were diagnosed as pyogenic spondylitis complicated by sepsis. The patient began receiving intravenous antimicrobial therapy that consisted of cefepime (CFPM) (2 g twice daily), vancomycin (VCM) (1 g twice daily), and clindamycin (CLDM) (600 mg four times daily), which commenced 15 minutes after the patient arrives at the hospital. However, he developed tachycardia and hypotension three hours after arrival at our hospital, so he received a blood transfusion and noradrenaline for serious shock vital and underwent emergent surgical open drainage since percutaneous drainage was difficult to perform because of scattered abscesses.

**Figure 2:**
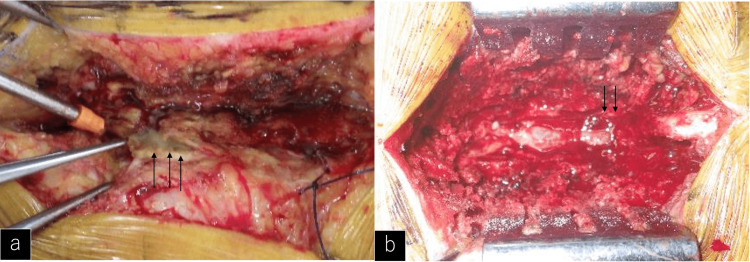
Intraoperative findings. a: Surgical site after splitting the lumbodorsal fascia white-colored pus was observed (black arrows). b: Surgical site after laminectomy. Necrotic adipose tissue was present around the dural sac (black arrows).

Incision of the lumbodorsal fascia allowed white-colored pus to drain from the paraspinal muscles (Figure 2a), and necrotic fatty tissue was observed around the dural sac (Figure 2b). Because of a large volume of bleeding during the surgical approach, the epidural vein was enlarged and coagulated. Platelets and fresh-frozen plasma transfusions were also administered during the procedure because of the patient’s low platelet count and coagulation dysfunction.

**Figure 3:**
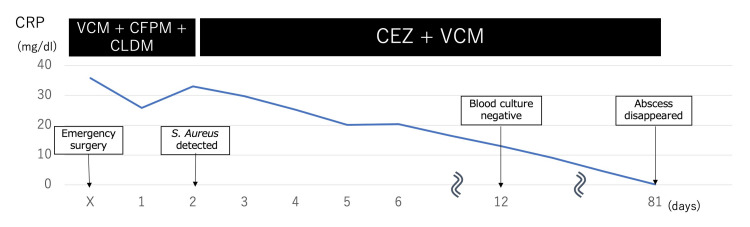
Antimicrobial therapy and C-reactive protein (CRP) after arrival at the hospital. The patient’s antimicrobial agents were changed after the identification of the causative organism. Thereafter, there was neither increase in CRP nor a recurrence of signs and symptoms, and antimicrobial therapy was continued for 81 days after surgery when the abscess was found to have disappeared. CRP: C-reactive protein, VCM: Vancomycin, CFPM: Cefepime, CLDM: Clindamycin, CEZ: Cefazolin, *S. Aureus*: *Staphylococcus aureus*

On the first postoperative day, the patient’s vital signs became stabilized, and noradrenaline was discontinued. On the second postoperative day, the organism in the blood culture was identified as methicillin-susceptible *Staphylococcus aureus*, and the CFPM was changed to cefazolin (CEZ) 2 g four times a day intravenously. Blood samples were taken for culture every three days and turned negative 12 days after surgery. An MRI performed 81 days after surgery appeared to show the resolution of the abscess, and intravenous antimicrobial therapy was stopped (Figure 3).

MMT of the proximal lower extremities was recovered to grade 5 bilaterally in the iliopsoas muscles and 4 bilaterally in the quadriceps muscles. But MMT of the distal lower extremities showed poor recovery in the tibialis anterior muscles (grades 2 right and 1 left) and 0 bilaterally in the extensor digitorum longus, flexor digitorum longus, and gastrocnemius muscles. Three years after surgery, the patient remains free of recurrence with improvement in the activity of daily living to the extent that he could walk for approximately 20 minutes with the use of a short leg brace.

## Discussion

Although there are no guidelines for the treatment of pyogenic spondylitis complicated by sepsis, there are reports of good outcomes achieved by treatments that were based on the practice guidelines for the treatment of sepsis [3]. The guidelines for sepsis state that an early diagnosis of sepsis by the SOFA or the quick SOFA followed by lifesaving measures that include resuscitation are the highest priorities [2]. Recommendations include the initiation of broad-spectrum antimicrobial therapy after specimens are obtained for culture, and attempts to control the infected lesions should be performed without the use of invasive procedures. Echo-guided and CT-guided drainage have been found to be less invasive and more effective [3]. Invasive surgery is recommended for the treatment of intra-abdominal infections and soft tissue infections after the patient's general conditions improve [2,3].

In our patient, the quick SOFA was used to make a prompt diagnosis of sepsis upon his arrival at our hospital, and antimicrobial agents were started after blood culture specimens were obtained. Even after antimicrobial agents were started, the patient's vital signs worsened, which included decreased blood pressure and worsening tachycardia; and the patient received support for his hypotension. After physical examination showed findings consistent with a spinal lesion, we found multiple small abscesses on MRI. Urgent open drainage was performed to control infection since a puncture was difficult to perform for drainage because of small and multiple abscesses.

Rutges et al. reported that *S. aureus* is the most common causative organism of pyogenic spondylitis, and has high rates of complication and mortality [4]. They recommended eight weeks of antimicrobial therapy for the treatment of *S. aureus* pyogenic spondylitis. Their findings suggest that pyogenic spondylitis caused by *S. aureus* is more frequent than pyogenic spondylitis due to other organisms, and is more likely to be severe. For our patient, we planned to administer antimicrobial therapy for six weeks after his blood cultures became negative. However, resolution of the abscesses was not achieved, and 81 days of antimicrobial therapy were required until the abscesses disappeared.

The duration of therapy should be determined based on diagnostic imaging. Although it has been reported that the addition of surgical treatment does not improve the long-term prognosis [4], the cases of Rutges et al. that required surgical treatment may have been more severely affected than the patients who were treated conservatively [4]. The impact of surgery on outcomes warrants further study.

Early surgical intervention is recommended for cases of pyogenic spondylitis that are resistant to conservative treatment or are complicated by severe bone destruction, neuropathy, or spinal instability [5-7]. Anemia, hypoalbuminemia, multiple intervertebral lesions, and epidural abscesses have been reported as factors associated with resistance to conservative treatment [8]. Our patient showed neuropathy, hypoalbuminemia, and an abscess in the epidural space; findings that were consistent with the recommendation for emergent surgical treatment. However, since previous reports have not reported detailed vital signs, we believe that it is essential to take the acute phase treatment of the disease to the individual patient.

Additionally, with regard to pyogenic spondylitis, a previous study on the outcomes of patients with epidural abscesses found that neurological outcomes were significantly better when interventional treatment was initiated within 36 hours and significantly worse with diffuse lesions [9]. The same study also reported that two of 27 patients died, and only 40% of the patients had resolution of their signs and symptoms even with appropriate therapeutic intervention, which is a very poor study outcome [9]. The risk factors for death included older age, medical comorbidities, elevated white blood cell counts, low platelet counts, low albumin levels, septic shock before and after surgery, cardiac arrest, pneumonia, multiple blood transfusions, and the presence of motor dysfunction [10]. Most of these factors are associated with the presence of chronic comorbidities and the patient's poor condition, making it difficult to intervene after the onset of the condition. With regard to motor dysfunction, however, making an appropriate diagnosis immediately after the onset of the disorder is very important, because that allows early treatment and may improve outcomes. Our patient’s risk factors included problems with mobility apparent four days before surgery and disturbed intestinal motility. Septic shock developed on the day of surgery. Although the patient had risk factors for death and poor functional prognosis because several days elapsed from the onset of his signs and symptoms to intervention, we were able to save the patient’s life.

Risks of the recurrence of the condition include a history of intravenous drug injection, disordered intestinal motility at presentation, and local infection after surgery [11]. Our patient did not develop recurrence, and his general condition and motor paralysis improved over time.

Although emergency surgery is sometimes recommended in septic shock, many physicians hesitate to perform spine surgery because of the invasive procedure and the difficulty in accessing the lesion. However, there are cases that can be saved through collaboration among spine surgeons, anesthesiologists, intensive care physicians, and infectious disease physicians.

There is a concern when cases similar to our case are encountered. Our patient's vital signs were controlled with the use of vasopressor agents, but preoperative judgment is extremely important in difficult-to-control cases because surgical invasion can be lethal. In addition, the use of vasopressor agents in surgery and postoperative systemic management requires the cooperation of many specialists, including anesthesiologists, emergency room physicians, and infectious disease specialists. Thus there may be patients for whom the treatments we used would be difficult to administer in different hospitals based on the available specialties of each hospital.

## Conclusions

We treated a patient with pyogenic spondylitis complicated by septic shock, who was saved by emergency surgery and comprehensive treatment including antimicrobial administration.

The outcome of our patient suggests that surgery may be a lifesaving measure in cases whose uncontrollable vital signs by pyogenic spondylitis are complicated by sepsis. Preoperative judgment is extremely important in difficult-to-control cases because surgical invasion can be lethal.
